# Trends in advanced maternal age pregnancies in Brazil: a time-series study (2011–2021)

**DOI:** 10.1590/1806-9282.20250897

**Published:** 2025-12-05

**Authors:** Enzo Bogucheski Ribeiro Machado, Ricardo Pinal de Mello, Thais Francielle de Souza, Ariane Giansante Souza, Mariane Nunes de Nadai, Ênio Luis Damaso

**Affiliations:** 1 Universidade de São Paulo, Faculdade de Medicina de Bauru – Bauru (SP), Brazil.; 2 Universidade de São Paulo, Hospital de Reabilitação de Anomalias Craniofaciais – Bauru (SP), Brazil.; 3 Universidade Nove de Julho – Bauru (SP), Brazil.

**Keywords:** Advanced maternal age, Brazil, Live births, Time factors, Pregnancy, Epidemiology

## Abstract

**OBJECTIVE:**

The aim of the study was to assess national trends in advanced maternal age pregnancies in Brazil between 2011 and 2021 and discuss their implications for maternal health care planning.

**METHODS:**

This ecological time-series study analyzed data from the Brazilian Live Birth Information System between 2011 and 2021. Births among women aged ≥35 years were identified, and temporal trends were evaluated using segmented regression models (joinpoint regression) to estimate Annual Percent Change and Average Annual Percent Change, both nationally and across the five Brazilian macro-regions.

**RESULTS:**

A sustained increase in pregnancies among women of advanced maternal age was observed nationwide. From 2011 to 2019, the Annual Percent Change in Brazil was 5.3%, followed by a slower rise from 2019 to 2021 (Annual Percent Change=2.4%). The most pronounced growth was seen among women aged ≥45 years after 2016 (Annual Percent Change=6.4%). Regionally, the highest Average Annual Percent Changes were observed in the Central-West (5.5%), Northeast (5.2%), and North (5.0%), reflecting geographic variations in the pace and timing of this demographic shift. The overall Average Annual Percent Change for Brazil was 4.7%, confirming a consistent upward trend over the study period.

**CONCLUSION:**

The rising prevalence of advanced maternal age in Brazil reflects broader demographic and social trends. Public health strategies must adapt to this reality, strengthening prenatal care and risk management protocols for older mothers.

## INTRODUCTION

Pregnancy at advanced maternal age (AMA), typically defined as occurring in women aged 35 years or older^1^, has become increasingly common in both developed and developing countries. This trend reflects demographic and sociocultural changes, including increased female participation in the workforce, higher educational attainment, greater access to contraceptive methods, and shifts in reproductive life planning^1,2^. In Brazil, this pattern parallels a nationwide decline in fertility rates, accompanied by a rising proportion of births among women aged 35 and above^3^.

While delayed childbearing may represent important social and professional achievements, it also poses significant clinical challenges. Advancing maternal age negatively affects oocyte quality and quantity, alters the endometrial environment, and reduces the functional reserve of several physiological systems, particularly cardiovascular, renal, and endocrine^4,5^. As a result, AMA pregnancies are associated with an increased risk of complications such as gestational diabetes, hypertensive disorders of pregnancy, premature rupture of membranes, cesarean delivery, and postpartum hemorrhage^6-8^.

From a perinatal perspective, AMA is also linked to higher risks of prematurity, intrauterine growth restriction, low birth weight, fetal death, congenital anomalies, and prolonged neonatal intensive care unit (NICU) admissions^9,10^. Recent cohort studies and meta-analyses have shown that women over the age of 40 are up to twice as likely to experience adverse fetal and neonatal outcomes, even after adjusting for sociodemographic and obstetric confounders^11,12^. Furthermore, AMA has been associated with increased rates of severe maternal morbidity and mortality, particularly in settings with limited access to specialized obstetric care^13^.

Time series analysis in the field of maternal and child health allows for the identification of epidemiological patterns over time, enabling the detection of trends, seasonal variations, and the potential impacts of public health interventions. By assessing changes in the distribution of maternal outcomes, this methodological approach contributes to evidence-based planning, resource allocation, and health policy decision-making. Such a strategy is particularly relevant in resource-limited settings, where optimizing care delivery is essential to improve maternal and perinatal outcomes^14^.

In this context, understanding the epidemiological trends of AMA is essential for informing maternal health policy and resource allocation. This study aimed to analyze national and regional trends in AMA pregnancies in Brazil between 2011 and 2021, using data from the Brazilian Live Birth Information System (SINASC).

## METHODS

This is an ecological time-series study based on secondary data of live births in Brazil between January 2011 and December 2021. The primary objective was to assess temporal trends in the prevalence of pregnancies in women aged 35 years and older (AMA) in Brazil and its regions from 2011 to 2021^1^.

Data were obtained from SINASC, a publicly available and nationally standardized database maintained by the Brazilian Ministry of Health, which contains comprehensive information on all registered live births in the country. As the data are de-identified and aggregated, the study was exempt from informed consent and did not require approval from a Research Ethics Committee, in accordance with Brazilian regulations (National Health Council Resolutions No. 466/2012 and No. 510/2016).

All births to women aged ≥35 years during the study period were included. The rate of live births among women aged 35 years or older was calculated by dividing the number of live births to mothers aged ≥35 years by the total number of live births in the same year, and multiplying the result by 100. The 11-year time frame (2011–2021) was chosen to allow the identification of long-term patterns in the incidence of AMA pregnancies and potential inflection points. In addition to national-level data, the analysis was stratified by the five official geographic regions of Brazil: North, Northeast, Southeast, South, and Central-West. The dataset was cleaned and structured using Microsoft Excel^®^, and initial visualizations were developed using R software (version 4.3.1).

To assess temporal changes in the prevalence of pregnancies at AMA, we applied segmented regression analysis using the Joinpoint Regression Program (version 4.3.1.0), developed by the US National Cancer Institute. This statistical method models time-series data by fitting a sequence of linear regression segments connected at statistically determined inflection points, known as joinpoints. The method assumes piecewise linearity and identifies significant changes in slope, allowing for detection of periods with different rates of increase or stabilization^15^.

Joinpoint regression analysis was initially applied to evaluate temporal trends in the prevalence of births among women aged 35 years or older in Brazil. Stratified analyses were subsequently performed for maternal age subgroups (35–39, 40–44, and ≥45 years), with categories defined according to the distribution of live births to ensure sufficient sample size and statistical reliability. Additional analyses were conducted across the five Brazilian macro-regions (North, Northeast, Southeast, South, and Central-West) to explore geographic differences. Further stratifications were avoided to prevent the formation of subgroups with low case counts, which could affect the robustness and interpretability of the estimates.

For each identified segment, the Annual Percent Change (APC) and its 95%CI were calculated, representing the annual rate of change in prevalence. The Average Annual Percent Change (AAPC) was also estimated to summarize overall trends throughout the study period. Statistical significance was assessed using the Monte Carlo permutation method, and segmented Poisson regression models with log link and offset for the number of live births were employed, following the methodology used in the Joinpoint Regression Program developed by the US National Cancer Institute. Model selection was guided by the Bayesian Information Criterion (BIC) to reduce overfitting risk.

In addition to trend analysis, we also conducted a descriptive assessment of key maternal and neonatal characteristics by calculating their national mean proportions over the 11-year period. These included mode of delivery (cesarean or vaginal), maternal educational attainment, gestational age at delivery, birth weight, 1st and 5th min Apgar scores, and the prevalence of congenital anomalies. These complementary indicators were included to provide a comprehensive epidemiological profile of pregnancies at AMA in Brazil.

## RESULTS

Between 2011 and 2021, a total of 31,702,562 live births were recorded in Brazil, of which 4,361,675 (13.8%) occurred in women aged 35 years or older. When stratified by age, the average was 10.94% among women aged 35–39 years, 2.69% among those aged 40–44 years, and 0.17% among those aged 45 years or older. Average rates of live births in this age group varied across regions: 9.15% in the North, 11.76% in the Northeast, 16.18% in both the Southeast and South, and 12.80% in the Central-West. Detailed data can be found in the Supplementary file.

**Figure 1:**
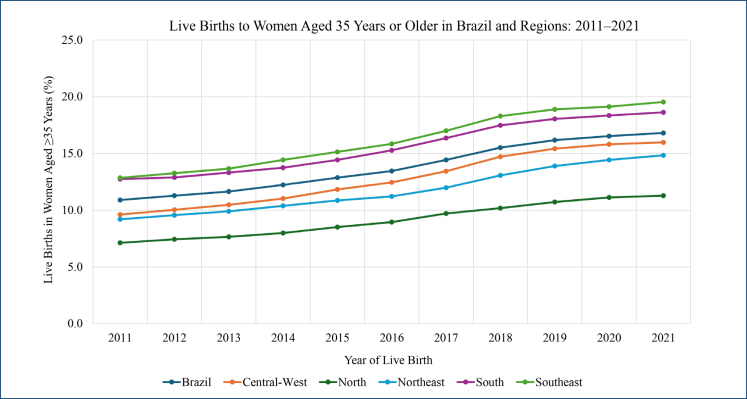
Proportion of live births to women aged 35 years or older in Brazil and its regions (Central-West, North, Northeast, South, and Southeast) from 2011 to 2021.

A progressive increase in the proportion of births among women aged 35 years or older was observed in Brazil, rising from 10.9% in 2011 to 16.8% in 2021. This upward trend was consistently observed across all maternal age subgroups—35–39, 40–44, and 45 years or older—indicating a steady growth in the prevalence of births in this age group throughout the study period. Figure 1 illustrates this temporal trend in Brazil and its five geographic regions—North, Northeast, Southeast, South, and Central-West—between 2011 and 2021. A consistent increase was observed in all regions, with the Southeast and South presenting the highest proportions, reaching nearly 20% by the end of the series. The North region maintained the lowest rates throughout the period, although it also followed an upward trajectory.

Most pregnancies were singleton (96.7%) and reached full term (84.9%≥37 weeks), with a high prevalence of cesarean sections (~70%). Low birth weight (<2,500 g) occurred in 10.8% of cases. Apgar scores between 8 and 10 were reported in 84.9% at the first minute and 95.8% at the 5th min. Congenital anomalies were present in 0.87% of newborns, most commonly involving the musculoskeletal system. Regarding maternal education, 33.8% of women had completed at least 8 years of schooling.

Temporal trends were analyzed using joinpoint regression to detect significant changes in trend patterns. APCs were calculated for each segment, and the AAPC summarized the overall trend from 2011 to 2021.

Joinpoint regression analysis demonstrated a statistically significant increase in the proportion of births among women of AMA between 2011 and 2021. The overall AAPC was 4.7% (95%CI 4.2–5.6), reflecting a sustained upward trend. Among women aged 35–39 years, the AAPC was 4.4% (95%CI 4.0–4.9), while the 40–44 and ≥45-year groups showed AAPCs of 5.4% (95%CI 5.2–5.7) and 3.7% (95%CI 3.1–4.4), respectively. The most marked acceleration occurred in the 40–44 age group from 2015 onward (APC=7.3%). These results underscore a demographic shift in maternal age and are detailed in Table 1.

**Table 1:** Annual Percent Change and Average Annual Percent Change in the prevalence of pregnancies at advanced maternal age by age group, Brazil, 2011–2021.

Age group	Period	APC (95%CI)	AAPC (95%CI)
Total	2011–2019	5.3% (4.2 to 9.4)*	4.7% (4.2 to 5.6)*
	2019–2021	2.4% (-0.7 to 5.4)	
35–39 years	2011–2019	5.4% (5.0 to 6.1)*	4.4% (4.0 to 4.9)*
	2019–2021	0.7% (-1.5 to 3.3)	
40–44 years	2011–2015	2.7% (1.4 to 3.6)*	5.4% (5.2 to 5.7)*
	2015–2021	7.3% (6.9 to 7.9)*	
≥45 years	2011–2016	1.2% (-2.6 to 2.8)	3.7% (3.1 to 4.4)*
	2016–2021	6.4% (4.9 to 10.0)*	

APC: Annual Percent Change; AAPC: Average Annual Percent Change; CI: confidence interval.

* Statistically significant (p<0.05).

Table 2 presents the APC and AAPC values for the prevalence of live births among women aged 35 years or older across Brazil's regions from 2011 to 2021. In the Central-West, there was an increasing trend from 2011 to 2019 (APC 6.4%), followed by a deceleration from 2019 to 2021 (APC 2.2%), with an overall AAPC of 5.5%, indicating sustained growth over the period. In the North, an upward trend was observed in both segments (APC 5.5 and 2.9%), with an AAPC of 5.0%. In the Northeast, a joinpoint was identified in 2015, with growth trends in both the initial (APC 4.2%) and later segments (APC 5.8%), and an overall AAPC of 5.2%. The South showed an increase until 2019 (APC 4.9%), followed by a period of stabilization (APC 1.9%), resulting in an AAPC of 4.3%. The Southeast displayed a similar pattern, with an upward trend until 2019 (APC 5.2%) and stabilization thereafter (APC 1.8%); the overall AAPC was 4.5%. In summary, all Brazilian regions demonstrated an increasing trend in the prevalence of births among women aged 35 years or older over the study period, with variations in intensity and timing of deceleration.

**Table 2:** Annual Percent Change and Average Annual Percent Change in the prevalence of pregnancies in women aged 35 years or older, by geographic region, Brazil, 2011–2021.

Region	Period	APC (95%CI)	AAPC (95%CI)
Central-West	2011–2019	6.4% (5.7 to 12.0)*	5.5% (4.8 to 6.8)*
	2019–2021	2.2% (-1.8 to 5.8)	
North	2011–2019	5.5% (4.8 to 8.4)*	5.0% (4.6 to 5.7)*
	2019–2021	2.9% (0.6 to 5.3)*	
Northeast	2011–2015	4.2% (1.8 to 6.7)*	5.2% (4.2 to 6.1)*
	2015–2021	5.8% (4.6 to 7.0)*	
South	2011–2019	4.9% (3.9 to 5.9)*	4.3% (2.8 to 5.8)*
	2019–2021	1.9% (-5.8 to 10.4)	
Southeast	2011–2019	5.2% (4.7 to 8.5)*	4.5% (4.0 to 5.4)*
	2019–2021	1.8% (-1.2 to 4.6)	

APC: Annual Percent Change; AAPC: Average Annual Percent Change; CI: confidence interval.

* Statistically significant (p<0.05).

## DISCUSSION

Over the past decade, Brazil has experienced a consistent rise in the proportion of births among women of AMA, both at the national level and across all five macro-regions. Between 2011 and 2021, the national rate increased from 10.9 to 16.8%, signaling a demographic shift toward older maternal age. Temporal trend analysis using joinpoint regression confirmed this growth. The APC reflects the average annual variation within each segment, while the AAPC summarizes the overall trend across the entire period. In Brazil, the AAPC was 4.7%, indicating sustained growth in this age group. Among the subgroups, women aged 40–44 years showed the most significant increase (AAPC 5.4%), particularly after 2015 (APC 7.3%). Although births among women aged ≥45 years were less frequent, this group also exhibited a recent acceleration. Regional analyses revealed the highest AAPCs in the Central-West (5.5%), Northeast (5.2%), and North (5.0%), with trend inflection points around 2015 or 2019. These results suggest a national pattern of delayed motherhood, with regional differences in the intensity and timing of trend deceleration.

This phenomenon aligns with broader social and demographic transformations in Brazil, including increased female participation in higher education and the labor market, postponement of childbearing for professional and personal reasons, and expanded access to assisted reproductive technologies^14,15^.

Pregnancies at AMA are associated with elevated risks for both mother and newborn. Numerous studies have reported higher rates of gestational diabetes, hypertensive disorders, cesarean delivery, and postpartum hemorrhage among older mothers^16,17^. From a perinatal perspective, there is also increased risk of preterm birth, low birth weight, NICU admissions, and congenital anomalies^18^. A recent Brazilian study found that women over 40 had twice the risk of adverse neonatal outcomes, even after adjusting for confounders^19^.

This rise in AMA pregnancies brings not only clinical concerns but also broader implications for healthcare systems. The growing demand for obstetric care tailored to older maternal age raises questions about the adequacy of health system preparedness. It is essential to evaluate whether healthcare resources are being strategically allocated to accommodate this demographic shift, particularly in terms of prenatal surveillance, maternal-fetal medicine services, and access to assisted reproductive technologies. Moreover, the increasing prevalence of AMA underscores the need for continuing education and specialized training for healthcare professionals, ensuring they are equipped to manage the more complex clinical scenarios associated with older pregnant patients. This includes managing higher rates of comorbidities, the use of advanced diagnostic tools, and delivering patient-centered counseling on risks and care options. Without strategic planning and investment, the growing burden of AMA pregnancies could strain health services and exacerbate existing inequities in maternal care^16-19^.

Although all regions exhibited rising trends in AMA pregnancies, the pace and timing varied. The Southeast and South maintained the highest proportions throughout the study period, possibly reflecting greater access to education, reproductive technologies, and health services. In contrast, the North and Northeast—regions, historically marked by higher fertility and lower socioeconomic indicators—had lower initial proportions but showed sharp increases after 2015. The Central-West showed a more pronounced rise in the early part of the series (2011–2019), possibly linked to rapid urbanization and improvements in healthcare access^20^.

These disparities highlight the importance of considering regional sociodemographic contexts in fertility and reproductive health analyses. Brazil is marked by significant interregional inequalities in income distribution, education, infrastructure, and urbanization^21,22^, all of which may influence reproductive trajectories and access to maternal healthcare services. Public policies should be tailored to these diverse realities, ensuring equitable care for women of AMA across different geographic and social settings.

The impact of public health emergencies should also be considered. Public health emergencies may have influenced reproductive behavior across age groups. For example, the rise in AMA births observed after the Zika virus outbreak (2015–2016) coincides with a documented decline in fertility among younger women during that period^23^. Similarly, the slight deceleration in AMA trends between 2019 and 2021 may reflect indirect effects of the COVID-19 pandemic, such as reduced access to reproductive health services and heightened maternal risk perceptions^24^. However, these interpretations remain speculative, as our study design and data do not allow for causal inference.

Social and racial inequalities must also be acknowledged. Black and Indigenous women in Brazil experience disproportionately higher rates of maternal morbidity and mortality, even within a universal health system, revealing persistent structural barriers to quality care^25^. Thus, reproductive risks in AMA should be interpreted not only from a biological perspective, but also within the broader sociostructural framework that shapes maternal and neonatal health outcomes.

This study offers a comprehensive national and regional overview of AMA pregnancy trends in Brazil; however, it presents inherent limitations. The use of secondary data from the SINASC system, although standardized and nationally representative, is susceptible to underreporting and potential misclassification of some key variables. In addition, individual-level determinants such as infertility, access to assisted reproductive technologies, or reproductive intentions were not captured, limiting causal inference.

Importantly, comparisons with younger maternal age groups (such as women aged 18–34) were not included, as they fell outside the predefined scope of the study. The primary objective was to investigate temporal trends and regional disparities in births among AMA women, rather than to evaluate relative risks across age groups. Therefore, the analysis was purposefully restricted to this population. Future research should consider incorporating comparative age-group analyses, including racial and ethnic profiles, gestational characteristics, and neonatal outcomes, to provide a more detailed understanding of how AMA intersects with other social determinants of maternal health.

In conclusion, the results underscore an ongoing demographic and epidemiological transition in Brazil, marked by a sustained rise in births among women of AMA. This shift calls for maternal health strategies that move beyond clinical risk management and encompass social determinants, regional disparities, and structural inequalities. Future studies are essential to deepen the understanding of this phenomenon, particularly the individual and contextual factors driving delayed childbearing. Research should address the long-term health outcomes for mothers and children, the impact of infertility and access to assisted reproductive technologies, and the intersection between reproductive trajectories and socioeconomic status. Moreover, qualitative investigations may help capture women's perspectives, motivations, and reproductive intentions. Evaluating the effectiveness of current public policies and exploring targeted interventions, especially in underserved regions, will be critical to ensure that maternal care is equitable, evidence-based, and responsive to the changing reproductive landscape.

## CONCLUSION

Between 2011 and 2021, Brazil experienced a marked increase in pregnancies among women of advanced maternal age, reflecting broader demographic, social, and epidemiological shifts. This trend underscores the need for health policies that are not only clinically appropriate but also socially responsive and regionally sensitive. The observed impact of public health crises such as the Zika outbreak and the COVID-19 pandemic on reproductive behavior highlights the vulnerability of maternal health to external shocks. These findings reinforce the urgency of strengthening equitable healthcare systems and developing targeted strategies to support women of AMA across diverse social and geographic contexts.

## Data Availability

The datasets generated and/or analyzed during the current study are available from the corresponding author upon reasonable request.
